# Dezocine prevents sufentanil-induced cough during general anesthesia induction: a meta-analysis of randomised controlled trials

**DOI:** 10.1186/s12871-020-01076-w

**Published:** 2020-06-22

**Authors:** Zhencheng Xiong, Ping Yi, Jipeng Song, Mingsheng Tan

**Affiliations:** 1 Graduate School of Peking Union Medical College, Chinese Academy of Medical Sciences, Beijing, 100730 People’s Republic of China; 2 Department of Spine Surgery, China-Japan Friendship Hospital, No. 2 Yinghua Dongjie, Hepingli, Chaoyang District, Beijing, 100029 People’s Republic of China

**Keywords:** Dezocine, Sufentanil, Cough, General anesthesia, Randomised controlled trials

## Abstract

**Background:**

Sufentanil is one of the opioids currently used to induce general anesthesia, and cough is one of the most common complications. Many drugs have been used to prevent sufentanil-induced cough (SIC), and dezocine is one of them. Dezocine is an analgesic, acting as partial antagonist of κ-receptors and agonist of μ-receptors. The purpose of our meta-analysis is to evaluate the efficacy of dezocine on SIC.

**Methods:**

We searched multiple databases including PubMed, Embase, ScienceDirect, the Cochrane Library, and China National Knowledge Infrastructure databases (CNKI) to identify studies that met the inclusion criteria. This meta-analysis focused on the incidence and severity of SIC after dezocine intervention, as well as adverse effects. This meta-analysis was registered on PROSPERO with reference number ID: CRD 42020144943.

**Results:**

Five randomised controlled trials (RCTs) were identified, including 890 patients. Each study was a comparison of dezocine with an equal volume of 0.9% saline. When the injection dose of dezocine was 0.1 mg/kg, the incidence (pooled risk ratio (RR) = 0.03, [95% CI: 0.02 to 0.07], *P* < 0.00001, *I*^*2*^ = 0%) and severity (mild: RR = 0.07, [95% CI: 0.03 to 0.18], P < 0.00001, *I*^*2*^ = 0%; moderate: RR = 0.05, [95% CI: 0.02 to 0.16], P < 0.00001, *I*^*2*^ = 0%; severe: RR = 0.04, [95% CI: 0.01 to 0.16], P < 0.00001, *I*^*2*^ = 0%) of SIC were significantly decreased. There were no statistically significant differences in vital signs between the two groups based on the results of the pooled analysis.

**Conclusion:**

This meta-analysis showed that dezocine significantly reduced the incidence and severity of SIC in the induction of general anesthesia, but had no significant effect on vital signs. More high-quality RCTs are needed to complement existing conclusions.

## Background

Opioids are widely used in the induction and maintenance of general anesthesia, mainly through the action of opioid receptors (μ, κ, δ), and opioids have a strong analgesic effect, fast onset, short duration, and reduction of cardiovascular response [1]. The most commonly used opioids in general anesthesia include fentanyl, sufentanil and remifentanil [2]. Due to different doses, routes of use, and population, opioids may have a series of adverse effects, such as nausea, vomiting, cough, addiction, urinary retention, and even respiratory depression [2]. Studies have shown that the incidence of cough caused by fentanyl, remifentanil, and sufentanil was 32% [3], 27.6% [4], and 28.5% [5], respectively. Coughing caused by the application of sufentanil is called sufentanil-induced cough (SIC) [6]. According to previous studies, independent risk factors for SIC include aging, body weight, smoking, injection time of opioid, and a priming dose of vecuronium, regardless of gender, the presence of either bronchial asthma or chronic obstructive pulmonary disease, or prior use of atropine [7].

SIC is generally transient, self-limiting, and benign in general anesthesia [8, 9]. According to the number of coughs recorded, the severity of cough was divided into mild (1–2), moderate (3–5) and severe (> 5) [6]. Studies have shown that SIC occurs mostly within 1 min of intravenous injection of opioids [6, 10, 11]. However, cough increases intracranial, intraocular and intra-abdominal pressures, which can cause a series of adverse effects [8]. SIC is more dangerous for patients with comorbidities such as increased intracranial pressure, brain hernia, brain trauma, cerebral aneurysm, increased ocular pressure, open eye injury, arterial aneurysm resection, a full stomach, pneumothorax, or hypersensitive airway disease [10]. It is necessary to take effective measures to suppress the occurrence of SIC in general anesthesia.

Many research teams had taken pharmacological or nonpharmacological measures to prevent SIC. Among them, nonpharmacological measures include slowing down the injection rate, diluting the drug concentration, reducing the drug dose, using the peripheral injection site, verifying the proper administration sequence of the drug, and instructing the patient to perform the huffing maneuver [12–15]. Pharmacological measures include ephedrine [16], dezocine [6, 10, 11, 17, 18], clonidine [19], ketamine [20], dexamethasone [20], and lidocaine [4, 9, 16].

Dezocine is a mixed agonist-antagonist opioid that is structurally similar to pentazocine [1]. Recent studies have shown that dezocine is an antagonist of κ-receptor and a partial agonist or antagonist of μ-receptor [1]. In recent years, several studies had shown that the administration of dezocine effectively prevented the occurrence and reflex degree of SIC in general anesthesia induction [6, 10, 11, 17, 18]. Moreover, the inhibitory effect of dezocine is related to the injected dose, and the effect is best when the dose is 0.1 mg/kg [21]. Dezocine, a member of opioids, also has similar side effects, such as postoperative nausea and vomiting (PONV), respiratory depression, and prolongation of anesthesia recovery time [10, 11]. Therefore, in clinical practice, we should also pay attention to the potential adverse effects of dezocine. We conducted a meta-analysis of randomised controlled trials (RCTs) to assess the efficacy of dezocine on SIC in order to provide a reference for clinical practice.

## Methods

We carried out this meta-analysis according to the Preferred Reporting Items for Systematic Reviews and Meta-Analyses (PRISMA) statement [22].

### Search strategy

In order to obtain all the literature related to our research, first of all, two researchers independently used the keywords combined with free words to search multiple databases according to Cochrane Collaboration guidelines, such as PubMed (1966 to May 1, 2020), Embase (1980 to May 1, 2020), ScienceDirect (1980 to May 1, 2020), Cochrane library (1966 to May 1, 2020), and CNKI (1980 to May 1, 2020). Next, potentially related literature was searched from a list of references in all included studies. We searched for the following terms “dezocine”, “sufentanil-induced cough or SIC”, “general anesthesia”, “sufentanil”, and “opioid” with the Boolean operators “AND or OR” by using Medical Subject Headings (MeSH) terms and corresponding keywords. Then, two researchers independently screened the above retrieved literature by reading the titles and abstracts. Finally, the selected literature was further filtered by reading the full text. After the discussion, all disagreeable literature was resolved.

### Study selection

All trials included in our study meet the following criteria: (1) All patients included in these RCTs had an American Society of Anesthesiologists (ASA) physical status classification of I–II and scheduled for elective surgery under general anesthesia; (2) All included studies were original RCTs; (3) In all included studies, two groups were given either intravenous dezocine 0.1 mg/kg or a matching placebo (equal volume of 0.9% saline); (4) None of the patients received any premedication in all included studies; (5) The incidence and severity of cough for 2 min after opioids injection were recorded in all included studies; (6) The full text of the included literature can be obtained, and the measurement data of incidence and severity of SIC, systolic blood pressure (SBP), diastolic blood pressure (DBP), heart rate (HR), and pulse oximeter oxygen saturation (SpO_2_) can be extracted.

Following studies were excluded from the meta-analysis: nonrandomized studies; the patients with a history of chronic cough, any sign of upper respiratory infection, asthma, smoking, clinical evidence of a difficult airway, bronchodilator or steroid therapy, use of pain medication (opioids or other drugs); studies not suitable with the inclusive criteria; and articles for which we were unable to obtain the full text and relevant data for pooled analysis.

### Data extraction

Data were extracted independently by two researchers. After discussion, disagreements in the data extraction process were resolved, and then another researcher used the spreadsheet to collect the data. We extracted the following data: first author, publication year, country, study type, number of participants (dezocine: placebo), Weight (kg), age, gender, ASA physical status I/II, intervention (dezocine: placebo), application method, the time of intervention earlier than anesthesia induction, the time of coughing after opioid injection, and outcomes data.

### Quality assessment

The risk of bias in each included RCT was assessed according to the Cochrane Handbook for Systematic Reviews [23]. The evaluation of bias can be divided into 7 sections: random sequence generation, allocation concealment, blinding of participant and personnel, blinding of outcome assessment, incomplete outcome data, selective reporting, and other bias. Each section can have a high risk of bias, low risk of bias and unclear risk of bias depending on the actual content of the included study [23].

### Statistical analysis

Different studies compared dezocine and placebo groups according to the incidence and severity of SIC, as well as side effects. We pooled and calculated data for the same outcome measure in all studies and placed them on the same form. The severity was divided into subgroups according to the classification. We analyzed dichotomous data using risk ratio (RR) and their 95% confidence interval (CI), such as the incidence and severity of SIC [2]. And we analyzed continuous data using weighted mean differences (WMD) and their 95% CI, such as SBP, DBP, HR, and SpO_2_. Statistical heterogeneity was calculated by using a chi-square test and *I*^*2*^ test [24]. It is considered that the *I*^*2*^ values of 25, 50, and 75% indicate low, moderate and high heterogeneity, respectively [25]. When *I*^*2*^ > 50%, *P* < 0.1, we performed a random-effect model; otherwise, a fixed-effect model was performed. If necessary, a sensitivity analysis was conducted to identify the origins of the significant heterogeneity. The funnel plot was often used to assess publication bias. In the current meta-analysis, publication bias and meta-regression were not assessable because they were usually only performed when at least 10 studies were included in the meta-analysis. The meta-analysis was performed using RevMan 5.3 for Windows (Cochrane Collaboration, Oxford, UK). If the result of the meta-analysis was a probability of *p* < 0.05, it was considered to be statistically significant.

## Results

### Study selection

**Fig. 1:**
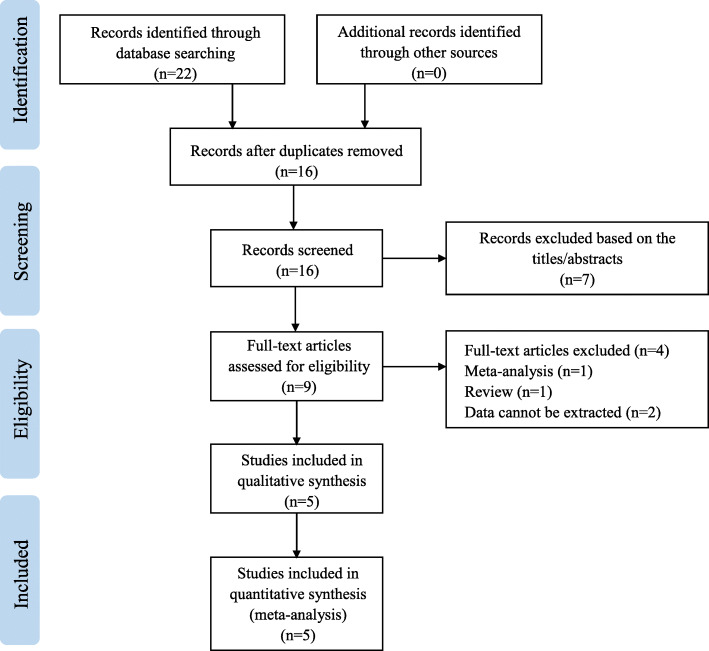
Flow chart of the selection of studies

Firstly, we searched in multiple databases by using keywords and free words, and finally confirmed 22 records. Then, a total of 9 records were screened out by reading titles and abstracts to remove duplicate records and irrelevant records. According to the inclusion criteria, records of non-RCT, letter or review, and records for which data could not be extracted were excluded. Finally, by reading the full text, a total of 5 RCTs were selected. Figure 1 showed the search strategy and the process of the study selection [6, 10, 11, 17, 18].

### Study characteristics

This meta-analysis included a total of 5 RCTs published between 2014 and 2017. Characteristics of all the studies included in the meta-analysis are shown in Table 1. All studies compared the effect of dezocine on the incidence and severity of SIC compared with placebo. In these studies, the number of patients in the dezocine group was the same as that in the placebo group [6, 10, 11, 17, 18]. In four studies, the number of male patients (408 patients) was greater than the number of female patients (382 patients) [6, 10, 11, 18]. The dose and time of sufentanil injection in the five studies were different (0.3 μg /kg, 5 s; 0.5 μg/kg, 3 ~ 5 s; 0.3 μg /kg, 5 s; 0.5 μg/kg, 3 s; 0.5 μg/kg, 3 s) [6, 10, 11, 17, 18]. Table 1 shows that all patients included in 5 RCTs had an ASA physical status classification of I–II and scheduled for elective surgery under general anesthesia. All patients were randomly assigned to receive either dezocine 0.1 mg/kg or a matching placebo (equal volume of 0.9% saline) [6, 10, 11, 17, 18]. In all studies, the time interval between the intervention and the opioid injection was different. However, after the injection of opioids, the time to start recording cough was consistent, both at 2 min [6, 10, 11, 17, 18].

**Table 1:** Characteristics of the studies included in the meta-analysis

Study	Country	Study type	Opioid	Opioid injection dose and time	Group	Weight (kg)	Age (years)	Gender M: F	ASA I/II	Intervention	Time 1	Time 2
Li et al., 2017 [10]	China	RCT	sufentanil	0.3 μg /kg, 5 s	Dezocine	62.2 ± 11.9	42.8 ± 11.5	80 (47/33)	55 /25	0.1 mg/kg, intravenous	10 min	2 min
					Placebo	64.1 ± 13.8	43.5 ± 10.4	80 (45/35)	56 /24	equal volume of 0.9% saline	10 min	2 min
Sun et al., 2015 [11]	China	RCT	sufentanil	0.5 μg/kg, 3 ~ 5 s	Dezocine	58 ± 9	51 ± 10	80 (36/44)	50 /30	0.1 mg/kg, 3 ~ 5 s, intravenous	3 min	2 min
					Placebo	59 ± 9	50 ± 9	80 (41/39)	55 /25	equal volume of 0.9% saline	3 min	2 min
Wang et al., 2015 [17]	China	RCT	sufentanil	0.3 μg /kg, 5 s	Dezocine	NP	NP	NP	NP	0.1 mg/kg, intravenous	10 min	2 min
					Placebo	NP	NP	NP	NP	equal volume of 0.9% saline	10 min	2 min
Liu et al., 2015 [6]	China	RCT	sufentanil	0.5 μg/kg, 3 s	Dezocine	62 ± 9	52 ± 13	185 (91/94)	125/60	0.1 mg/kg, 3 ~ 5 s, intravenous	2 min	2 min
					Placebo	60 ± 8	53 ± 11	185 (99/86)	117/68	equal volume of 0.9% saline	2 min	2 min
Xu et al., 2014 [18]	China	RCT	sufentanil	0.5 μg/kg, 3 s	Dezocine	61.2 ± 10.9	41.2 ± 14.3	50 (26/24)	NP	0.1 mg/kg, intravenous	5 min	2 min
					Placebo	62.1 ± 9.7	39.7 ± 16.1	50 (23/27)	NP	equal volume of 0.9% saline	5 min	2 min

*RCT* randomised controlled trial, *M* male, *F* female, *ASA* American Society of Anesthesiologists, *NP* not provided, *Time 1* record the time of intervention earlier than anesthesia induction, *Time 2* start recording the time of coughing after opioid injection

### Risk of Bias

**Fig. 2:**
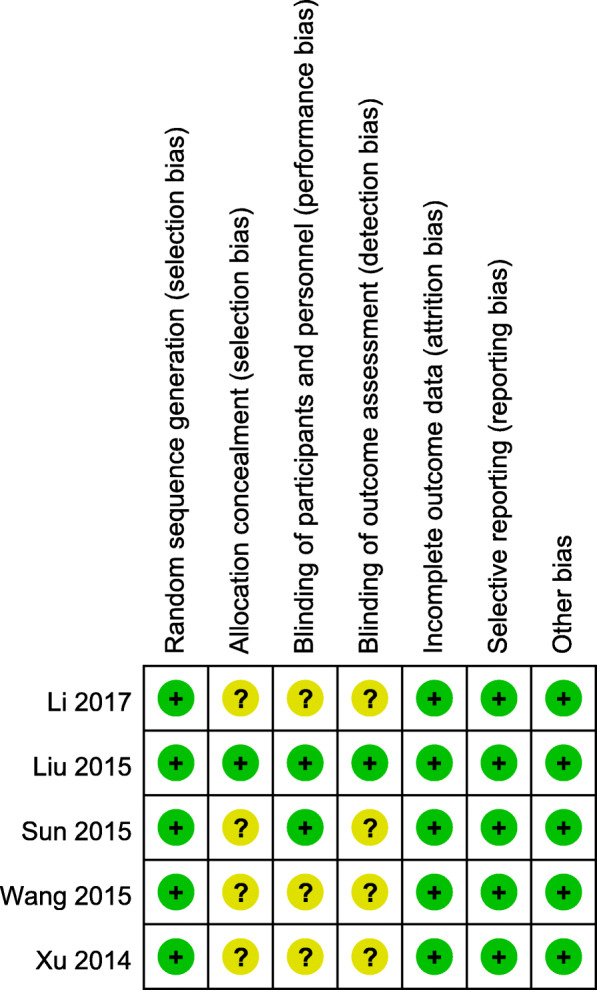
Summary of Risk of Bias Assessment

All five studies were considered to have a low risk of bias. Random sequence generation was found in 5 studies [6, 10, 11, 17, 18]. Allocation concealment and blinding of outcome assessment were found in one study [6]. Blinding of participants and personnel was found in 2 studies [6, 11]. As shown in Fig. 2, none of the five studies found incomplete results data, selective reports, and other bias [6, 10, 11, 17, 18].

### Results of the meta-analysis

After carefully reading and analyzing the included articles, we summarized the evaluation tools used to measure the effect of patients after receiving dezocine or placebo treatment, including the incidence and severity of SIC, SBP, DBP, HR, and SpO_2_. Among them, the incidence of SIC is the primary outcome measure.

#### The incidence of SIC

**Fig. 3:**
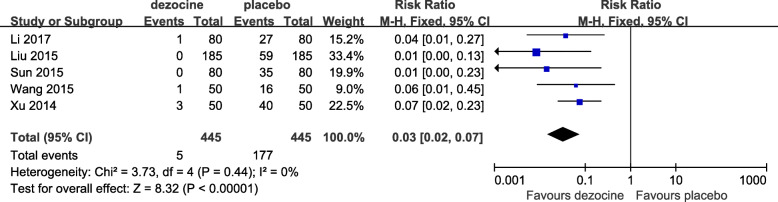
Forest plot of the effect of dezocine compared with placebo on the incidence of SIC (SIC, sufentanil-induced cough)

Five RCTs used the incidence of SIC as the primary outcome measurement [6, 10, 11, 17, 18]. As shown in Fig. 3, the forest plot shows the effect of dezocine on the incidence of SIC compared with placebo. A total of 5 studies (890 patients) provided data on the incidence of SIC for the dezocine and placebo groups [6, 10, 11, 17, 18]. In 2 studies, no cough occurred in the dezocine group 2 min after sufentanil injection [6, 11]. In all included studies, cough occurred 2 min after sufentanil injection in the placebo group [6, 10, 11, 17, 18]. Based on the results of the pooled analysis, there was a statistically significant difference between the two groups at the incidence of SIC (RR = 0.03, 95% CI: 0.02 to 0.07, *P* < 0.00001, *I*^*2*^ = 0%) (Fig. 3). When *I*^*2*^ = 0%, it means that the three studies are highly homogenous, and the summary analysis results of the data are meaningful. The *P* value indicates that dezocine significantly inhibits the occurrence of SIC compared with an equal volume of 0.9% saline.

#### The severity of SIC

**Fig. 4:**
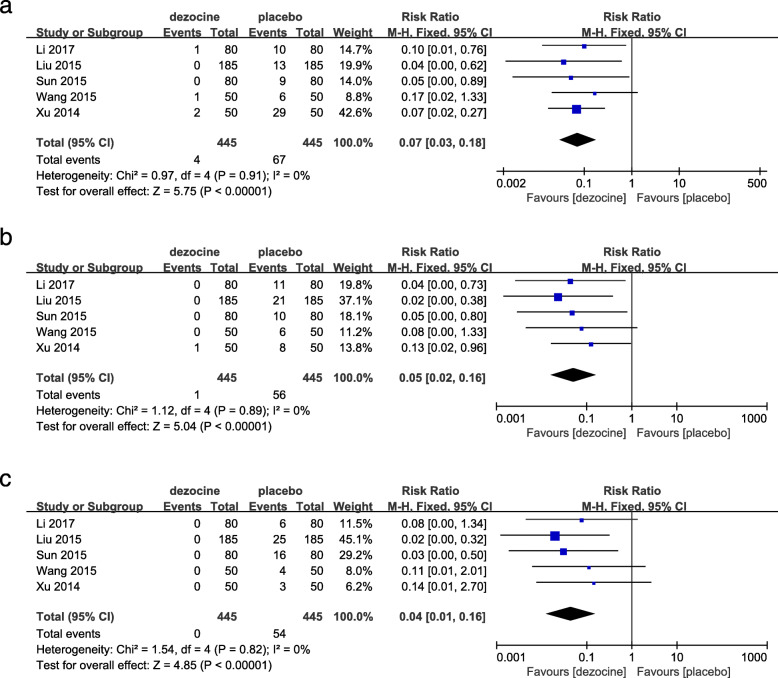
Forest plot of the effect of dezocine compared with placebo on the severity of SIC. The severity of SIC was graded by cough frequency (**a**. mild (1–2), **b**. moderate (3–4), **c**. severe (≥5))

Five RCTs used the severity of SIC as the secondary outcome measurement [6, 10, 11, 17, 18]. As shown in Fig. 4, the forest plot shows the effect of dezocine on the severity of SIC compared with placebo. The severity of SIC was graded by cough frequency as mild (1–2) (Fig. 4a), moderate (3–4) (Fig. 4b), and severe (≥5) (Fig. 4c) [6]. A total of 5 studies (890 patients) provided data on the severity of SIC for dezocine and placebo groups [6, 10, 11, 17, 18]. In the dezocine group, there were 4 cases of mild SIC, 1 case of moderate SIC, and no coughing of severe SIC [6, 10, 11, 17, 18]. In the placebo group, 67 patients were in the mild SIC, 56 patients were in the moderate SIC, and 54 patients were in the severe SIC [6, 10, 11, 17, 18]. Based on the results of the pooled analysis, there was a statistically significant difference between the two groups in the severity of SIC (mild: RR = 0.07, [95% CI: 0.03 to 0.18], *P* < 0.00001, *I*^*2*^ = 0% (Fig. 4a); moderate: RR = 0.05, [95% CI: 0.02 to 0.16], P < 0.00001, *I*^*2*^ = 0% (Fig. 4b); severe: RR = 0.04, [95% CI: 0.01 to 0.16], P < 0.00001, *I*^*2*^ = 0% (Fig. 4c)). The above results indicated that dezocine not only significantly inhibited the occurrence of SIC, but also reduced the severity of SIC compared to an equal volume of 0.9% saline.

#### Adverse effects

**Fig. 5:**
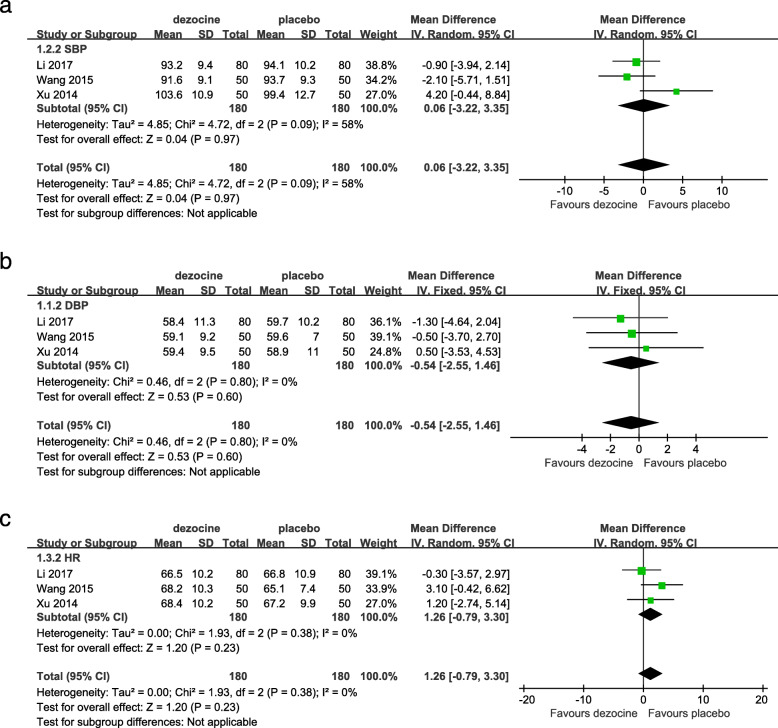
Forest plot of the effect of dezocine compared with placebo on SBP (**a**), DBP (**b**), HR (**c**) after general anesthesia. (SBP, systolic blood pressure; DBP, diastolic blood pressure; HR, heart rate)

As shown in Fig. 5, the forest plot shows the effect of dezocine on the vital signs compared with placebo. A total of 4 studies (730 patients) reported hemodynamic changes in SBP, DBP, and HR [6, 10, 17, 18]. However, only one study (160 patients) provided data on SpO_2_ for dezocine and placebo groups [11]. We deleted one of the four studies because it provided the highest blood pressure and HR value 2 min after induction of anesthesia [6]. If the obvious changes in vital signs due to the application of dezocine or placebo affect the safety of anesthesia, it is called adverse effects [2]. We performed a pooled analysis of vital sign data after general anesthesia to compare the dezocine and placebo groups. After induction of anesthesia, there was no statistically significant difference in post-intervention vital signs between the two groups based on the results of the pooled analysis (SBP: MD = 0.06, [95% CI: − 3.22 to 3.35], *P* = 0.97, *I*^*2*^ = 58% (Fig. 5a); DBP: MD = -0.54, [95% CI: − 2.55 to 1.46], *P* = 0.60, *I*^*2*^ = 0% (Fig. 5b); HR: MD = 1.26, [95% CI: − 0.79 to 3.30], *P* = 0.23, *I*^*2*^ = 0% (Fig. 5c)). The *P* value indicated that the vital signs are still at approximately the same level after the intervention in both groups. This result illustrates the effect of dezocine on vital signs, which is not significantly different from an equal volume of 0.9% saline. When *I*^*2*^ > 50%, this means that the included studies are highly heterogeneous. The heterogeneity of the above results is high and may be related to the inclusion of too few studies, requiring more high-quality RCTs.

Because only one study provided data on SpO_2_ for dezocine and placebo groups, a pooled analysis could not be successfully performed. In one study, dizziness, lethargy, and respiratory depression were reported in the dezocine group [10]. In the future, more high-quality RCTs are needed to assess the adverse effects of dezocine.

### Publication bias

The funnel plot is often used to assess publication bias but is usually only performed when at least 10 studies are included. Five RCTs were included in this meta-analysis. Therefore, in the current meta-analysis, publication bias and meta-regression cannot be fully evaluated.

### Sensitivity analysis

If necessary, a sensitivity analysis was conducted to identify the origins of the significant heterogeneity. Due to the high heterogeneity of SBP and HR before and after treatment, we performed a sensitivity analysis to assess the reliability of the results. However, there were only three studies that met the inclusion criteria, and the reliability of the results might be affected by the limited number of studies included.

## Discussion

Opioids are primarily used in pain management due to morphine-like effects [1]. Opioids have good analgesic and sedative effects and are commonly used for the induction and maintenance of general anesthesia [6]. At the same time, opioids have many common side effects, including cough, in the induction of general anesthesia. The essence of cough is a defensive airway reflex [26]. Cough receptors are located in epithelial cells that are sensitive to both mechanical and chemical stimuli [26]. Among opioid-induced cough, SIC is not uncommon [6, 10]. The current mechanism for the occurrence of SIC is still controversial. Previous related studies have differed in the dose, injection rate or injection order of opioids, as well as pretreatment with different drugs during induction of general anesthesia, which can reduce SIC [2, 6]. Sufentanil, a thienyl analog of fentanyl, primarily activates μ-opioid receptors and also induces coughing during anesthesia induction like fentanyl [3, 27]. Studies have shown that fentanyl enhances the excitability of rapidly adapting receptors, and stimulates the release of histamine and neuropeptides in the airways to cause coughing [28]. Recent studies have shown that fentanyl-induced cough may contain a pulmonary chemoreflex mediated by rapidly adapting receptors (irritant receptors) or vagal C-fiber receptors (J-receptors) located in proximity to pulmonary vessels [29].

Studies have shown that SIC is generally transient, self-limiting, benign in general anesthesia, and more dangerous for patients with comorbidities such as increased intracranial pressure, arterial aneurysm resection, a full stomach, or hypersensitive airway disease [8–10]. The occurrence of SIC may bring challenges to the induction of general anesthesia, affecting the safety of patients. Therefore, there have been many studies in taking certain measures to prevent the occurrence of SIC [2]. The measures taken are generally divided into two types, pharmacological and nonpharmacological measures. Nonpharmacological measures are primarily achieved by modulating the dose and rate of opioid injection [21]. Pharmacological measures include a variety of drugs, including dexamethasone, lidocaine commonly used in local anesthesia, and even dezocine, which is also an opioid [2, 6]. Dezocine, a mixed opioid receptor partial agonist/antagonist, is an opioid that is structurally similar to pentazocine [1]. In previous studies, it was highly controversial whether dezocine was an agonist or antagonist of the kappa receptor. In the latest research, Liu et al. [1] found that dezocine is a partial mu receptor agonist, a kappa receptor antagonist, and has two new molecular targets (norepinephrine transporter, NET; and serotonin transporter, SERT). Studies have shown that dezocine effectively inhibits the incidence and severity of SIC [6, 10, 11, 17, 18].

Our meta-analysis summarizes the RCTs of dezocine in the prevention of SIC in general anesthesia induction. The indicators analyzed included the incidence and severity of SIC, as well as changes in vital signs. The pooled analysis showed that dezocine significantly inhibited the incidence and severity of SIC compared with an equal volume of 0.9% saline. All included studies recorded coughs occurring 2 min after opioid injection. We also compare changes in vital signs after the intervention. The pooled analysis also showed no difference in the changes in vital signs compared with an equal volume of 0.9% saline. However, the current specific mechanism for the prevention of SIC by dezocine is still unclear, and more relevant research is still needed.

### Limitations

This meta-analysis still has some limitations. First, most studies lacked details of random sequence generation, allocation concealment, blinding of participant and personnel, and blinding of outcome assessment. Second, the injection dose and time of opioids were not exactly the same. Third, the time for starting the injection of dezocine is not uniform, the recording time of vital signs is not uniform, and the injection dose is single. Fourth, all trials were from the same country and might have an impact on the conclusions. Finally, the number of studies that met the inclusion criteria was very limited. Therefore, more high-quality RCTs need to be invested in the future.

## Conclusions

The results of the above analysis indicated that the injection dose of 0.1 mg/kg of dezocine significantly inhibited the incidence and severity of SIC. There was no significant difference in the effects of dezocine on SBP, DBP, and HR compared with placebo. A number of publications have summarized the effects of dezocine on fentanyl-induced cough, but there are too few articles on SIC. This meta-analysis is the first to evaluate the efficacy of dezocine on SIC. However, more high-quality RCTs are needed to determine the optimal injection dose and time of dezocine in the future to supplement the existing conclusions.

## Data Availability

All data used in this review are included in this published article.

## Abbreviations

SIC: Sufentanil-induced cough
CNKI: China National Knowledge Infrastructure databases
RCTs: Randomised controlled trials
PONV: Postoperative nausea and vomiting
MeSH: Medical Subject Headings
PRISMA: Preferred Reporting Items for Systematic Reviews and Meta-Analysis
ASA: American Society of Anesthesiologists
SBP: Systolic blood pressure
DBP: Diastolic blood pressure
HR: Heart rate
SpO_2_: Pulse oximeter oxygen saturation
RR: Risk ratio
CI: Confidence interval
WMD: Weighted mean differences
M-H: Mantel-haenszel
IV: Inverse variance
